# Trauma, early life stress, and mindfulness in adulthood

**DOI:** 10.1186/s40359-024-01563-6

**Published:** 2024-02-14

**Authors:** Jonathan Gibson

**Affiliations:** South Dakota School of Mines and Technology, 501 E St. Joseph Street, Rapid City, SD 57701 United States of America

**Keywords:** Early life stress, Mindfulness, Interoception, Emotion regulation, Attachment, And alexithymia

## Abstract

This article is a review that was inspired by recent studies investigating the effects of childhood trauma or early life stress (ELS) and mindfulness in adulthood. One recent study found that some forms of abuse and neglect led to higher scores in several subscales of a self-report measure of mindfulness. The authors concluded that some forms of ELS can help cultivate certain aspects of mindfulness in adulthood. However, and in contrast to this recent finding, much of the extant literature investigating ELS and trauma are linked to emotional dysregulation, alexithymia, and a host of psychopathologies in adulthood which makes the results of this study surprising. Central to the mindfulness literature is cultivating an open, non-reactive, or non-judgment awareness of inner experiences which are important for emotional regulation. In this paper, I review some of the effects of trauma or ELS on critical neural circuits linked to mindfulness, interoception, attachment, and alexithymia which I hope may clarify some of the conflicting findings from this study and throughout the literature and provide additional context and a framework that may inform research investigating these two constructs going forward.

## Introduction

A recent study [1] set out to explore the link between early life stress (ELS) and trait mindfulness in adulthood. Trait mindfulness was defined as: “the awareness that emerges through deliberate attention in the present moment, with intention, without judgment, making the most of the current experience” [1 p. 2]. As the authors point out, there is a gap in the literature exploring these two constructs and little is known whether ELS can affect the latter or vice versa. There is a wealth of literature demonstrating the benefits of mindfulness and how mindfulness can help build resilience, manage stress and emotions, and improve the overall quality of life which might have an indirect effect on childhood trauma [2–4], but the authors claimed a study exploring the link between the two had yet been done. It is important to note that there is at least one prior study that has explored the link between childhood and lifetime trauma, mindfulness, PTSD, and dissociative symptoms. That study will be reviewed in a subsequent section below. The authors in this recent study [1] conducted a cross-sectional correlational design from a Brazilian public university and most of the findings corroborate prior research on ELS and mindfulness. That is, those who experienced less ELS tended to score higher in various facets of mindfulness, at least as it was measured by the Five-Facet Mindfulness Questionnaire (FFMQ) and the Childhood Trauma Questionnaire (CTQ).

However, the researchers found some forms of ELS may help cultivate certain aspects of mindfulness in adulthood. Specifically, participants in their study who experienced more emotional abuse, emotional neglect, and physical abuse scored higher in the subscale of “non-reactivity to inner experience” and those who experienced more emotional abuse, emotional neglect, sexual abuse, physical neglect, and physical abuse scored higher in “acting with awareness,” though those correlations were modest [1]. The non-reactivity facet of this assessment tool is believed to measure one’s ability to notice internal sensations and emotions and mindfully process those and let them pass without overreacting or being overrun by them. The “acting with awareness” is believed to measure engagement and awareness of the present moment rather than being distracted or on autopilot. This type of awareness and non-reaction or non-judgment of inner experiences are central facets to the mindfulness literature and are critical for emotional regulation and psychological well-being [1, 5–7]. This novel finding is provocative especially when compared to prior studies investigating ELS or trauma which have been consistently linked to emotional dysregulation and a host of psychopathologies in adulthood. The authors suggest that this specific population may have learned to cultivate emotional regulation even in the presence of childhood trauma [1].

The purpose of this article is to provide additional context and a framework that may help inform some of the conceptual and methodological baggage that shapes these constructs. This paper is not meant to be comprehensive, but I will review some of the effects of trauma or ELS on critical neural circuits that influence the development of mindfulness, interoception, attachment, and alexithymia and how those capacities appear to be fundamentally shaped by the relational environment, or as the authors described it as ‘bio-social functions’ [1]. It may be possible the results of the study [1] may be skewed due to construct conceptualization and methodological limitation. In short, it is hoped that this framework can shed light and help account for some of the inconsistent and even contradictory findings in the contemplative or mindfulness literature and help identify putative neurological target interventions in the clinical literature.

As the authors [1] highlight, ELS including emotional, physical, sexual abuse, and neglect is linked to a number of physical and psychological disorders [4, 8–10]. These negative outcomes are consistent and well documented throughout the literature. Moreover, ELS has been shown to influence the development of neural structures linked to emotion processing and memory, [10–14] attachment and relational patterns, [15–18] interoception (awareness of the internal state of the body), [5–7, 19–22] which is believed to be foundational to mindfulness [5, 22–26], and alexithymia [27]. It is generally accepted that ELS can lead to maladaptive or unhealthy emotional and behavioral responses and a myriad of psychological disorders in adult life [see 1 for review].

## Mindfulness

To begin, a close examination of the constructs described in this study is needed. Despite its growing popularity in the scientific community and society generally, mindfulness itself remains broadly defined and loosely conceptualized [26, 28–29]. Critics [28] have emphasized that mindfulness has become an umbrella term that characterizes a large number of practices, processes, and characteristics spanning acceptance, awareness, non-judgment and memory. The confusion surrounding mindfulness includes the problems of defining and measuring it. The capacity to be mindful is believed to be multi-faceted [28–30, 17]. Some have argued that attention to the present moment may be the single most critical aspect of mindfulness [30]. Others emphasize a particular attentional style. For example, Kabat-Zinn [31] argued that mindfulness is not just moment-to-moment awareness, but a specific type of awareness that includes an objective, non-reactive, non-judgmental, and open-heart. These definitions, however, still leave room for interpretation [see 28 for review].

In addition to its broad definition, sometimes researchers refer to mindfulness as a particular meditation – whether it is an open-monitoring meditation, breathing mediation, or body scan [28]. This is also problematic because each meditation produces different effects and requires different attentional styles [26, 32]. It is generally believed in the contemplative literature that attention regulation is the prerequisite for other beneficial outcomes to take place [5, 33]. However, each of those meditations have been categorized in different ways. For example, a mindfulness meditation is often referred to as ‘open-monitoring’ (OM) meditation which explicitly prescribes a mindful attentional style to both interoceptive and exteroceptive sensations, thoughts, and emotions [34, 35]. Breathing and body scan meditations have been categorized as a focused attention (FA) meditation [34–36]. Focused attention meditations involve focusing and maintaining attention on a single object such as one’s breath, heartbeat, or a mantra [33].

The semantic ambiguity in the meaning of mindfulness or mindful meditations has implications. Van Dam and his colleagues [28] argued that any study using the term mindfulness must be carefully scrutinized to accurately ascertain what type of “mindfulness” was involved. They also urged scientists, practitioners, and the media alike to move away from the broad use of the term mindfulness and more clearly specify exactly what practices and processes are being taught. That is, when formal meditation is used in a study, one ought to consider whether a mindful or open-monitoring meditation or a focused attention meditation was the target intervention. For instance, the mindfulness-based stress reduction (MBSR) program consists of multicomponent treatments and employs both FA meditative techniques (body scan and yoga) and an OM or mindfulness technique (sitting meditation). Yet, all of these interventions require different attentional styles which produce different effects [26, 37]. As Holzel et al., [33] point out, it is unclear what role “mindfulness” may play in the various, documented outcomes. These distinctions are critical because how mindfulness is operationalized will determine what is measured and how and those differences can vary from scale to scale [29].

Even though mindfulness has its roots in Buddhism, the scientific investigation of mindfulness has been shaped by Western scientific methodologies and assumptions. Grossman [29] argued that mindfulness, in the Buddhist tradition, is meant to cultivate “truths” about personal, lived experience which is a subjective phenomenon that is difficult to measure using traditional, Western methodologies. This effort is further problematized because the definition and measurement of mindfulness is enmeshed in a ‘complex web of historical, social, economic, political, and technological factors’ [29]. The mindfulness assessments themselves – even with good psychometric scores of reliability and validity – are often operationalized in different ways and those meanings (including the meaning of mindfulness itself), and can differ from scale to scale [38]. Furthermore, there are semantic ambiguities in assessment items which have led to questionable outcomes e.g., binge-drinking students score more “mindfully” than healthy controls or long-term mindfulness meditators [see 29 for review]. It has also been shown that various scales don’t often correlate highly with one another [38, 39]. All of these challenges measuring and defining mindfulness should cause one to remain cautious in interpreting results. Mindfulness remains a broadly defined and loosely conceptualized construct and the assessment tools may be too imprecise to ideally capture these nuanced abilities.

### Mindfulness, the insula, and interoceptive or salience network

Both the FA and OM mediations produce different neurological and functional effects, [26, 33] but there are important commonalities. Research has shown that all meditations included in mindfulness practices directly shape the anatomy and function of the insula and interoceptive network (IA) or salience network (SN) [23–26, 32, 40–45]. The IA/SN network spans various brain regions, which include the insular cortex, anterior cingulate cortex (ACC), the inferior frontal gyrus, and the sensorimotor cortex, but also presents multiple connections to the amygdala, hypothalamus, hippocampus, and brainstem [5, 23–26]. To be clear, a recent meta-analysis has shown that every meditation type including FA, OM, mantra, and loving/kindness meditations have been shown to modulate the insula in some way [32]. Furthermore, the insula is believed to be the only neural structure that is modified by any and all meditations [32]. As Fox and Cahn [32] point out, given that the insula is the hub for interoception, this finding shouldn’t be surprising as the body plays a central role in mindfulness practices [5–7, 23–26].

Studies have consistently shown that dispositional and trait mindfulness is linked with increased activity and cortical thickness in the insula [23–26, 42–45]. Friedel et al., [25] found that these neuroplasticity changes are true not only in adults but also in adolescents. The authors argue: “While evidence for anterior insula involvement in adult long-term meditator has been interpreted to indicate an effect of mindfulness meditation on insula structure and function, the current results suggest that structural development of the anterior insula may contribute to the development of dispositional mindfulness” (pp. 67). Indeed, many have argued that increased interoception and the neuroplasticity changes produced within the insula, ACC, and IA/SN network are foundational to developing mindfulness [5–7, 22–26]. Thus, a close examination of the functions of insula, ACC, and IA/SN circuits will prove useful here.

### Emotional awareness, regulation, and interoception

de Morales et al., [1] rightly point to emotional awareness and emotional regulation as central facets of mindfulness as it is a consistent theme throughout the literature. Emotion regulation is also at the heart of psychological well-being as it enables an individual to develop appropriate, flexible, and adaptable responses in adult life [1]. Emotion regulation begins with recognizing a stimulus and then establishing a meaning [1]. Studies have shown that effective emotional regulation appears to be at least partly dependent upon accurate interoception [45–47, 5]. Indeed, in Buddhist philosophy, the first pillar to develop mindfulness is to develop a sense of the body, which includes an awareness of momentary sensation while distinguishing sensation from conceptual thought [5, 48].

The insula, ACC, and IA/SN network have been shown to be essential circuits not only for emotional awareness, but awareness of the present moment [see 49 for review] – a salient facet in the mindfulness literature. Craig [49, 50] connected human awareness to emotional awareness and interoception. In his review, he discussed how all stimuli or sensations that are salient to the individual are ultimately represented by feelings which are crucial neuropsychological constructs that function as the currency of awareness [49]. The insula, ACC, and IA/SN network translate interoceptive signals into feelings and emotions. This framework isn’t new as early and modern theories of emotion have emphasized the importance of interoceptive feedback in emotional states and cognitive processes [21, 49–59]. For example, Damasio, [51] building off the work of William James, [52] argued that positive or negative emotional feeling states are associated with visceral and other bodily responses to certain situations and awareness of those are essential for affective, cognitive, and interpersonal processes.

Studies have shown that individuals who are more aware of their body – higher levels of interoceptive awareness – report more intense emotional experiences than those who are less aware [54–56]. This is important because emotional experiences appear be associated with individual differences in one’s ability to both generate and perceive subtle bodily changes [56–58]. Zaki et al., [58] demonstrated that the interoceptive network is highly engaged in emotional processing and that “emotional experience is intimately tied to information about internal bodily states” (p. 498). The insula has been shown to be the key region which integrates information from the body *via* lamina 1 spinothalamic and vagal afferent tracts [49]. Much of those body sensations projects ultimately into the posterior portion of the insula and somatosensory cortices and is re-represented in the mid and anterior portion of the insula which is then sent to the prefrontal regions bringing subtle, interoceptive sensations into awareness [5, 49]. The anterior portion of the insula provides a multilevel integrated meta-representation of the state of the entire body integrating body sensations and top-down processes into a broader context [49–51, 5].

There is a growing body of literature indicating that learning to accurately discern bodily signals through meditation and mindfulness can enhance one’s ability to understand one’s emotional state [23–25, 60–62]. Contemplative practices, including mindfulness, produce neuroplasticity changes within IA/SN circuits increasing interoception by bringing subtle interoceptive cues into awareness [23–26, 43–47]. The observed neuroplasticity changes within those circuits can explain how meditation and mindfulness enhance interoceptive sensations and emotional awareness [see 26 for review]. This increased awareness can then be used to develop adaptive strategies to regulate stress and improve well-being [5, 63].

To summarize, emotional awareness and effective emotional regulation appear to be dependent upon accurate interoception [45–47, 55–58]. Interoception is necessary for emotional awareness, and thus, interoception becomes a basis for engaging emotional processing. To be mindfully aware of interoceptive sensations and resultant emotions in a stable, non-reactive awareness in stressful situations is a central feature in the mindfulness literature [5–7, 63]. Mehling and his colleagues [63] point out, being able to mindfully accept body sensations may reduce the emotional impact of unpleasant ones. This capacity may also enable one to “listen” to emotion-related sensations that are central to insight and decision making rather than being “overrun” by them. This raises two important questions: do individuals who have suffered from various forms of ELS mindfully process interoceptive sensations and the emotional effects? And, in addition, can the assessment tools used accurately capture this refined, nuanced ability?

## Interoception and mindfulness

Interoceptive awareness and mindfulness are associated but distinct constructs in mind-body interactions [26, 63]. Attention regulation is a critical distinction in teasing these two constructs apart [26, 63]. For example, in some mindfulness practices there is no distinction between attention directed to interoceptive sensations, exteroceptive stimuli, or conscious thoughts [63]. This is relevant as several studies highlight different attentional styles (that is, how and where one focuses attention) elicit different neural responses [32, 45, 62]. In the interoception literature, the assessments tools often fail to distinguish between different attention styles [63]. For instance, some scales do not differentiate from anxiety or hypervigilant attentional style to interoceptive sensations and mindful and open-monitoring styles [63]. Training individuals to focus solely on interoceptive sensations does not necessarily imbue participants with knowledge on how to alter attentional style or mental habits commonly employed to avoid unpleasant sensations when they emerge [5, 6, 26, 63].

Dispositional mindfulness may promote more adaptive interoceptive attentional style and enhance or illuminate discriminative capacities related to various bodily sensations [5, 6, 26, 63]. That is, intentional mindful awareness may provide a safe focal point from which one can view various signals from the body. As Hanley et al., [6] argue: “awareness of bodily sensations and the evaluative or regulatory tendencies applied to such sensations are important determinants of emotional health” (p. 5). One way to investigate how some can develop the mindful capacity to be aware of and sift through various interoceptive and emotional processes in a non-reactive, non-judgmental manner is to examine the development of the insula, ACC, and IA/SN network through the biosocial functions [1], specifically the attachment relationship [18].

### Insula and attachment

There is extensive empirical evidence demonstrating that early childhood relationships and experiences, including ELS, directly shape the development of a number of brain circuits, including and especially the insula, ACC, and IA/SN network [15, 16, 18]. Investigating ELS from the attachment relationship should help clarify how ELS directly shapes mindfulness abilities in adulthood. Indeed, a recent study found attachment orientation seems to have a unidirectional and causal effect on mindfulness in adulthood [17]. That is, those who had insecure attachments in childhood due to neglect or other forms of ELS, were unable to cultivate trait mindfulness in adulthood.

de Morales et al., [1] aptly point to biosocial functions in their discussion section. Research has clearly demonstrated that early life experiences, including attachment patterns in childhood, have enduring consequences throughout the lifespan on emotional regulation [64–67]. Oldroyd et al., [18] point out that the insula and IA/SN neural circuits that are necessary for interoception and emotional regulation show protracted post-natal development. The architecture and function of these neural circuits are heavily shaped by early experiences and relationships. Some have even argued that normal brain development may be dependent upon a secure attachment [15, 68] which is characterized by sensitive, loving, and supportive relationships [68, 69].

Children with secure attachments who have formed a secure bond with their primary caregiver manage their anxiety and autonomic arousal with a degree of trust due to the caregiver’s consistent and attentive response to the child’s needs [18, 68, 69]. Those interpersonal experiences shape internal working models and the development of the neural circuits involved not only in relational processes, but also interoception, mindfulness, and emotional regulation. These processes will be further unpacked below. In summary, when a child feels loved, secure, and trust in their relationship with their caregiver, they will use the caregiver as a “secure base” from which to explore the environment and manage their stress response [69].

Several studies have shown that individual differences in attachment patterns are characterized by different neural responses to stress [see 18 for review]. When a parent avoids responding to or delays meeting the child’s immediate needs (e.g., neglect), or is inconsistent or only conditionally available, then the child may develop an insecure avoidant or anxious attachment pattern [18]. Insecure attachment orientation is typically conceptualized along two dimensions: anxious and avoidant [70]. Individuals with insecure avoidant or insecure anxious attachments show not only altered IA/SN networks, but these individuals also suffer from dysregulated hypothalamus-pituitary-adrenal (HPA) axis activity in response to stress across the lifespan [71, 72].

Stress regulation and interoception utilize many of the same anatomical pathways between the brain and body [49, 54, 73–75]. Trauma or ELS found in the attachment relationship directly shape the neural circuits that govern interoception and distress, both of which are necessary for emotional regulation. Furthermore, researchers [73, 74] identified a direct link from the sympathetic nervous system to the insula, ACC, and IA/SN network with specialized neurons within that network called Von Economo Neurons (VEN’s). These neurons are believed to be a cerebral representation of the autonomic nervous system [73, 74]. Interestingly, these neurons are only found in the IA/SN network [49, 73, 74] and the gut or enteric nervous system [53] and are believed to process and integrate emotion and behavior [49, 73, 74]. Research is reliably showing that ELS affects the development of the HPA axis, the insula, ACC, and IA/SN network, which, in turn, affects interoception, one’s ability to be mindful, and to regulate stress and emotion. Furthermore, trauma and ELS have been shown to affect both the strength of those interoceptive signals and how those signals are perceived [18]. Friedel et al., [25] argue that there should be increased emphasis on the insula, and the IA/SN network as these circuits not only play a critical role in maintaining emotion and self-regulation, but also provides a distinct construct with a measurable neurobiological imprint.

### Attachment, interoception, and non-reactivity

Attachment related processes have also been linked to insular anatomy and activity. Studies have shown that those with an avoidant or anxious attachment pattern have markedly lower insular volume and smaller surface areas than those with a secure attachment [76–79]. Furthermore, those with avoidant attachment patterns have decreased insular electrical activity compared to securely attached controls [78]. Oldroyd et al., [18] argue, insensitive, slow, inconsistent caregiving or rejection of the infant’s distress impairs the child’s ability to form accurate bodily representations because the infant must rely on caregivers’ responses to help shape and inform accurate interoceptive states.

The insula also plays a critical role in comparing feelings in the present moment with those of the past and anticipation of the future [80], which plays an important role in meta-memory processing [81]. This meta-memory process can explain why interoceptive predictions that are associated with trauma or ELS are often distorted [40] as the insula becomes unusually overactive in individuals who have experienced abuse or trauma [21, 49] or underactive in those who have been neglected [17, 18, 82]. Individuals with an anxious attachment pattern might overemphasize or exaggerate bodily cues leading to emotional distress and dysregulation. In contrast, those with avoidant attachment patterns might minimize or suppress bodily cues [17, 18]. “This means that the more avoidant a person’s attachment style, the less attention they paid to their bodily cues and the less they tended to trust those cues” [18 pp. 5].

### ELS and mindfulness

The result de Morales et al., [1] found that various forms of ELS might help cultivate increased awareness of and non-reactive response to inner experience (i.e., interoception) is surprising because a central facet to mindfulness is the ability to pause, increase awareness, and gain greater access to sensations and emotions without being overcome by those feelings [5–7, 63]. The hypothesis in the original study [1] was that those who experienced certain types of ELS, including neglect and several forms of abuse, may be more aware and less judgmental of bodily sensations. However, the authors [1] highlight those participants continued to react with “greater intensity to their inner experiences” [ p. 9] which they argued revealed a deficit in their coping or emotional regulation strategies [p. 9]. This raises important questions: Are those participants mindfully processing interoceptive sensations and emotions? Or did those who experienced heightened levels of ELS develop patterns similar to an insecure attachment style consistent with the anatomical and functional neural changes characteristic of those patterns?

Avoidant individuals have often been described in the literature as manifesting a disconnect between bodily cues and their physiological responses [82]. These individuals may present as if they were calm while in a distressing situation (e.g., mindful), when they simply dissociated from or suppressed those sensations in a non-reactive way [83, 84]. As Oldroyd and her colleagues [18] argue, those with avoidant attachment patterns have learned to either minimize or suppress those signals. It is also possible those participants developed alexithymia which is defined as an impaired ability to be aware of, explicitly identify, and describe one’s feelings [85]. Those participants may be unable to accurately perceive and identify interoceptive signals and use those to inform their emotional state.

A recent study investigated the effects of childhood trauma, attachment, addiction, and alexithymia [27]. The results of this recent study [27] corroborated prior studies which found alexithymia is a common result of childhood trauma or ELS. Moreover, alexithymia is now recognized as a key factor responsible for non-adaptive strategies of regulating emotions [27, 86]. Characteristics of alexithymia include (1) difficulty identifying feelings and distinguishing between feelings and bodily sensations of emotional arousal, (2) difficulty describing feelings toward other people, (3) externally oriented cognitive style, and (4) low perspective taking, as well as difficulty describing and understanding the emotions of others [86].

Interestingly, the authors [27] found that the strongest predictor for developing alexithymia in adulthood were insecure anxious and avoidant attachment patterns from childhood. Specifically, the authors [27] found that “avoidant attachment style has the strongest negative impact on the development of a strategy for affect regulation and general emotional development” [p. 9]. Conversely, studies have shown that those with a secure attachment have an inverse relation to alexithymia [see 27 for review]. Insecure avoidant attachment styles also demonstrate lower levels of trust in personal relationships, [87] trust in themselves, [88] and the insula has been shown to be the neural correlate for evaluating trustworthiness of others [49]. The insula is also believed to be a critical neural circuit linked to alexithymia [49]. Thus, an individual who has not developed trust in a loving caregiver has not learned to trust others or themselves, nor can they expect their body to give them reliable signals that inform their emotional state [18, 27]. Indeed, there is an extensive body of literature that has linked insecure attachment styles to alexithymia [see 27, 90–91 for review].

There is growing evidence that accurate interoception develops initially in the context of interpersonal relationships [18]. A child’s attachment relationship characterized by either a warm and responsive connection with the primary caregiver, or a distressing relationship characterized by trauma, neglect, or indifference inevitably shapes those neural circuits related to stress and interoception. “To the extent that a child’s bodily experiences are denied, devalued, ignored, or punished by parents, the child will find ways to avoid feeling them, and develop a distorted sense of interoception” [18 pp. 10].

de Morales et al., [1] point to the “biosocial” facet of cultivating emotional awareness, emotional regulation, and mindfulness. It appears that ELS and trauma disrupt the attachment system which creates a ripple effect. In concert with a large body of literature, increased awareness and effective emotional regulation appear to be dependent upon accurate interoception. Accurate interoception is shaped by early life experiences, including the attachment relationship. Accurate interoception and proper development of the insula, ACC, and IA/SN network has been shown to be foundational to developing mindfulness and emotional regulation. Trauma or ELS seems to lead to insecure attachments, alexithymia, and host of psychopathologies.

### Attachment and mindfulness

Some research has explored why attachment and mindfulness may be linked. Both constructs are linked to the same neural circuitry, and both contribute to a range of positive outcomes including mental health, and self and emotion regulation [89, 90]. Ryan et al., [89] suggested that mindfulness and attachment have a bi-directional relationship. They argued that attachment security fostered enhanced awareness and attentiveness to relational patterns while mindfulness was believed to increase one’s capacity for a secure relationship by cultivating an open, receptive attention to relationship partners. Stevenson et al., [17] has challenged that assumption as they found that attachment orientation seems to play a unidirectional, causal role in the development of mindfulness. The authors wrote: “the organization of the attachment system and inner working models, resultant of caregiver warmth and availability, not only influence the way in which we view ourselves and others, but also the capacity in which we attend to our experiences” (pp. 21). Their research indicates that attachment orientation comes first and can predict and affect the capacity for mindfulness in adulthood.

### Mindfulness, trauma, PTSD, and dissociation

As mentioned in the introduction, there was at least one prior study investigating childhood trauma and mindfulness. Specifically, the authors explored whether mindfulness traits (measured using the FFMQ) would mediate the relationship between childhood and lifetime trauma, PTSD, and dissociative symptoms [91]. The authors found an inverse relationship between mindfulness, trauma, PTSD, dissociative PTSD, and trauma-related altered states of consciousness (TRASC). That is, those who had increased traumatic experiences and PTSD symptomology had a decreased capacity for trait mindfulness. Unlike de Moralez et al., [1], this study did not find a relationship between trauma and the mindfulness facets of non-reactivity and acting with awareness. Moreover, the authors argued that a decreased capacity for different facets of mindfulness may be one mechanism by which trauma exposure leads to the development of PTSD or trauma-related distress or dissociation.

Interestingly, however, the authors did find that individual differences in mindfulness traits may partially mediate the association between increased lifetime and childhood trauma exposure and posttraumatic symptoms [91]. They found the facets of describing, acting with awareness, non-judgment, and non-reactivity revealed a negative relationship with trauma, PTSD, and PTSD symptomology. They did make particular note of the observing facet. They found observing was associated with increased PTSD symptomology and linked to childhood and lifetime trauma and exposure. The authors wrote: “Observing trait may be a risk factor *for*, rather than a protective factor *against*, mental health problems” (pp. 678).

This is interesting because some studies have found a link with the observing trait and a history of trauma [91, 92], while others have linked the observing trait with measures of good psychological health [93]. Herein lies a contradiction as some critics [see 28 for review] have pointed out. How can the observing trait be linked to both mindfulness and emotional regulation, while also linked to emotion dysregulation and a host of psychopathologies? Some hypothesize that the observing facet serves as a marker of vividness or depth of experience [91, 92]. Boughner et al., [91] wrote: “in persons exposed to life experiences that are for the most part positive, nurturing, and safe, being more mindfully observant will heighten the influence of such adaptive life experiences in encouraging psychological health. In contrast, if a person is repeatedly exposed to life events that are highly stressful or traumatic in nature, those who are predisposed toward heightened Observing may experience such events with increased intensity, increasing risk for aversive consequences” [pp. 677].

Early childhood experiences whether nurturing, safe, and secure, or traumatic, abusive, or neglectful alter internal working models and neural circuits linked to those functions that appear to have lifelong effects. Therefore, investigating mindfulness from a developmental and relational or attachment model may prove to be a useful framework in interpreting some of the inconsistent findings throughout the mindfulness and contemplative literature and help identify putative target interventions from a clinical perspective. Indeed, a recent study [94] has identified disruptions in the dorsal mid-insula across a number of psychological disorders, which the authors found were anatomically distinct from other brain regions in affective processing.

## Conclusion

I have attempted to lay out a conceptual or theoretical framework from which to interpret the link between trauma, ELS, and mindfulness. It is my hope this article can prove to be a useful reference piece in aiding future research. It may be possible that ELS or trauma can help cultivate certain aspects of mindfulness in adulthood. If this is the case, this finding warrants further, careful investigation. It is also possible that the results of this recent study [1] may be skewed due to several factors. Among those is the definition and conceptualization of mindfulness. How mindfulness is operationalized changes how it is measured [29]. Popular scales don’t often highly correlate and the meanings within those scales can differ [29, 38]. There are also semantic ambiguities in assessment items which have led to questionable outcomes such as binge-drinking students scoring higher in mindfulness than practiced meditators [29, 38]. Boughner et al., [91] also pointed to construct limitations as a potential confound in the literature. They highlighted that a significant overlap exists between some mindfulness assessments and PTSD diagnostic criteria in the DSM-5. For example, the mindful trait of describing “overlaps considerably with alexithymic symptomology of PTSD associated with the emotional numbing criteria of DSM-5 PTSD” [pp. 677]. They argue further that the non-reactivity trait implies the opposite of emotion dysregulation but may overlap with trauma-related immobilization defenses [91, 95], which is in concert with the conceptual framework of avoidant attachment and alexithymia described above.

de Moralez et al., [1] acknowledged the questionnaire as a limitation in their study and indicated that the questions “proved outdated, especially with those questions that started with the word “non” [p. 9]. The authors had to assist participants in answering the questions as some participants became fatigued during the process raising questions on the accuracy of the results [1]. Moreover, the authors noted that during data collection, stress levels were elevated for the given population due to a variety of factors. There are also a number of limitations using self-report assessments. For example, the use of questionnaires rather than interviews is believed to exaggerate the clinical significance of trauma-related symptoms in the general population [95]. Furthermore, trauma questionnaires measure only the occurrence of an event but not the frequency or severity [91]. Finally, it was reported that less than 20% of the participants in this study had a meditation or mindfulness practice [1]. This is relevant because a mindfulness meditation practice has shown to reliably produce neuroplasticity changes within the insula, ACC, and IA/SN network which could affect the results [see also 94]. However, mindfulness questionnaires do not always correlate with mindfulness meditation practices [96]. This variable ought to be explored more closely in the future.

The authors [1] also focused on emotion regulation. Emotional awareness is the first step to emotional regulation which requires recognition of a stimulus and to assign it meaning [1]. Key facets of emotional awareness and regulation appear to be dependent upon accurate interoception, [46–47, 54–58] and interoception is believed to be foundational to mindfulness [5, 22–26] as all three of these abilities utilize much of the same neural circuitry. Furthermore, a growing body of evidence indicates that accurate interoception is shaped by early life experiences. The effects of ELS and trauma on the nervous system is widely discussed in the literature and the results are consistently linked to emotional dysregulation and a host of psychopathologies [4, 8–10]. In short, trauma or ELS have been shown to affect a number of brain regions. The focus here has been on the insula, ACC, and the IA/SN circuits as they appear to be critical circuits in attachment, interoception, which appears to be necessary for emotional awareness and regulation, mindfulness, and alexithymia. As Friedel and his colleagues [25] argue, there should be increased emphasis on these regions because it provides a distinct construct with a measurable neurobiological imprint. Furthermore, novel treatments focused on the insula may aid in more effective interventions from a clinical perspective as a number of psychological disorders have been shown to have disruptive functions within the insula that are showing to be anatomically distinct from other brain regions [94].

Some have argued that the insula is an ‘underestimated region of the brain’ [97] while others have argued that it is still poorly understood [98]. This is interesting because the insula and IA/SN circuits are not only linked to the functions described above, but also implicated in all subjective feelings [49. 50]. That is, these circuits appear to be the cortical structures that not only engender interoception and emotional awareness, but awareness in the present moment [50].

Emotional awareness and regulation are also associated with individual differences in ability to both generate and perceive subtle bodily changes [57–58]. Those who have experienced ELS often develop insecure attachments with the characteristic anatomical and functional effects on those neural circuits [15–18, 76–79, 82–84]. Someone with an insecure anxious attachment style might overreact to internal sensations while an insecure avoidant may suppress or ignore those. Therefore, the result that some forms of ELS might lead to a non-reactive and non-judgment heightened awareness of inner experience is provocative [1]. Alternatively, and consistent with an extensive body of literature, it seems plausible that those who scored higher in the awareness and non-reactive or non-judgmental aspect of the assessment tool [1] may be suppressing or minimizing those signals rather than mindfully, non-judgmentally or non-reactively processing them [see also 94]. This pattern is consistent with the avoidant attachment styles [18, 82–84] and alexithymia [27, 49, 86–87, 99–100] which could account for the results of the study [1].

As the authors emphasized, [1] understanding the link between ELS and mindfulness should encourage researchers to explore the two more carefully. Their findings emphasize the importance of emotional regulation as ELS is consistently linked to emotional dysregulation and psychological disorders later in life, but their results suggest there may be some positives. The authors [1] also suggested that those within that specific population may have developed various strategies to improve emotional regulation and became more mindful of their internal states. Thus, a more careful, precise analysis of these constructs is needed. I applaud the authors for their study as it brings these important constructs into focal view.

## Data Availability

Not Applicable.
